# Surface Characterization, Antimicrobial Activity, and Biocompatibility of Autopolymerizing Acrylic Resins Coated with *Reynoutria elliptica* Extract

**DOI:** 10.3390/plants9101292

**Published:** 2020-09-29

**Authors:** Song-Yi Yang, Min-Kyung Kang

**Affiliations:** 1 Department and Research Institute of Dental Biomaterials and Bioengineering, Yonsei University College of Dentistry, Seoul 03722, Korea; syyang88@yuhs.ac; 2 Department of Dental Hygiene, Hanseo University, Chungcheongnam-do 31962, Korea

**Keywords:** coating resin, *Reynoutria elliptica* extract, contact angle, microhardness, polyphenol, antimicrobial activity, cell viability

## Abstract

We conducted surface characterization to assess the biocompatibility and investigate the antimicrobial activity against oral pathogens in autopolymerizing acrylic resins, coated with light-curable coating resin, containing various concentrations of *Reynoutria elliptica* extract (0, 200, 400, and 600 µg/mL). The *R. elliptica* extract powder was prepared using a freeze-drying technique. Further, a goniometer and microhardness tester were used to determine the water contact angle, and Vickers hardness, respectively; color measurements were performed on the uncoated and coated acrylic resin disks. The polyphenol content of the extracts from the coated acrylic resin disk was analyzed using UV-VIS spectroscopy. The antimicrobial activity of the coated acrylic resin disk against *Streptococcus mutans* and *Candida albicans* was observed for 24 and 48 h by measuring the optical density using spectrophotometry. In addition, biocompatibility was confirmed by testing the cell viability according to ISO 10993-5. The water contact angle, Vickers hardness, and color change values of the coated acrylic resin disks were not significantly different from the control. Polyphenol was detected in all experimental groups, with no significant differences between the experimental groups. The experimental groups exhibited significant antimicrobial activity against *S. mutans* and *C. albicans* compared to the control group, after 48 h of incubation. The cell viability between the control and experimental groups was not significantly different. The proposed coating resin containing *R. elliptica* extract is applicable on dental acrylic resins, due to their antimicrobial properties and excellent biocompatibility, with no deterioration of surface characteristics.

## 1. Introduction

Managing the hygiene of dentures or removable orthodontic appliances is a critical factor in maintaining the oral health of patients and achieving aesthetic goals [1,2]. The main component of dentures and orthodontic appliances such as retainers is polymethyl methacrylate (PMMA)-based acrylic resin, used in the majority of removable dental prosthetic devices, due to its ease of processing, molding, and favorable physicochemical properties [3,4,5]. However, patients who use dentures or removable orthodontic retainers often have poor oral hygiene and high infection rates of pathogenic microorganisms due to improper management of the acrylic resin based prosthetic devices [6,7,8,9,10]. *Streptococcus mutans* metabolizes acids by attaching to the surface of the tooth in the oral cavity. The acid generated demineralizes the tooth structure and causes dental caries [11,12]. *Candida albicans* also adheres to the surface of the denture or to the oral mucosa, causing diseases, such as denture stomatitis [13,14].

A biofilm inevitably forms on the surface of the denture that the colonized bacteria attaches to materials [15,16]. Therefore, it is important to suppress the growth of microorganisms that adhere to the dentures. This phenomenon applies to both denture surfaces and orthodontic acrylic resins [17,18,19,20]. Various approaches have been investigated to improve oral hygiene and prevent oral disease [21]. These include the use of denture cleaners, mechanical cleaning, denture base resins mixed with antimicrobial substances, and coating resins containing antimicrobial substances [22,23,24,25,26,27] However, the long-term use of denture cleaners can corrode the metallic materials, such as clasps and may impact the color stability of the denture base resin [28,29,30,31]. Mechanical cleaning can wear the surface of acrylic resins, creating a rough surface. However, this approach is patient-dependent because hygiene varies according to the cleaning habits of individual patients [32,33]. As antimicrobial ingredients are used in the acrylic resin, several components are released, which may induce the death of bacteria that cause oral disease. However, another research have postulated that the mechanical properties of dentures also deteriorate [34,35]. Therefore, it is important to apply an antibacterial coating that does not negatively impact the mechanical and physical properties of the dentures, while inhibiting the activity of oral pathogenic microbes and preventing oral disease [36].

The application of natural medicines is an effective and safe approach to inhibit the microbial activity of oral pathogens [37,38,39,40]. *Reynoutria elliptica* is a perennial plant of the *Polygonaceae* family that effectively treats diuretic and menstrual disorders, promoting blood circulation, alleviating pain, and relieving respiratory diseases [41]. In addition, *R. elliptica* is also widely studied as a suitable material, due to its antimicrobial and anti-inflammatory properties with minimal side effects, thereby making it applicable for prolonged use. Based on the previous research, the process of verifying the efficacy of *R. elliptica* using cells of oral origin is also necessary to extend the clinical application of plants to the dental field. As the anti-inflammatory effects of *R. elliptica* extract are evaluated using oral derived cells, it is important to select cells that can only prove the effects of the material [42,43]. Some studies have investigated the prevention of dental caries by including growth inhibitory components against *S. mutans*. In addition, *R. elliptica* demonstrated excellent antibacterial activity against *Helicobacter bacteria* [44,45].

However, previous studies were limited to testing antimicrobial activity using *R. elliptica* as the raw material, without examining how the composition of the coating resin impacts the surface. There have been no reports on the use of a naturally derived *R. elliptica* extract to induce surface changes in the denture materials and to observe the surface properties, and antimicrobial effects on oral pathogenic bacteria and fungi. Therefore, we explored the potential application of *R. elliptica* extract as a constituent of the surface coating resin for use as an antimicrobial dental material. The surface characterization of autopolymerizing acrylic resins, coated with light-curable coating resin, containing *R. elliptica* extract was conducted to assess the biocompatibility, and confirm the antimicrobial activity against oral pathogens. The first null hypothesis of this study states that the surface coating treatment with various concentrations of the *R. elliptica* extract does not significantly impact the water contact angle, hardness, and color of the acrylic denture material surface. The second null hypothesis is that the surface coating treatment with different concentrations of the *R. elliptica* extract does not show significant differences in antimicrobial activity against *S. mutans* and *C. albicans* strains. The third null hypothesis is that the surface coating resin containing various concentrations of the *R. elliptica* extract does not significantly influence the viability of L929 fibroblast cells.

## 2. Results

### 2.1. Water Contact Angle of the Coated Surface

**Figure 1:**
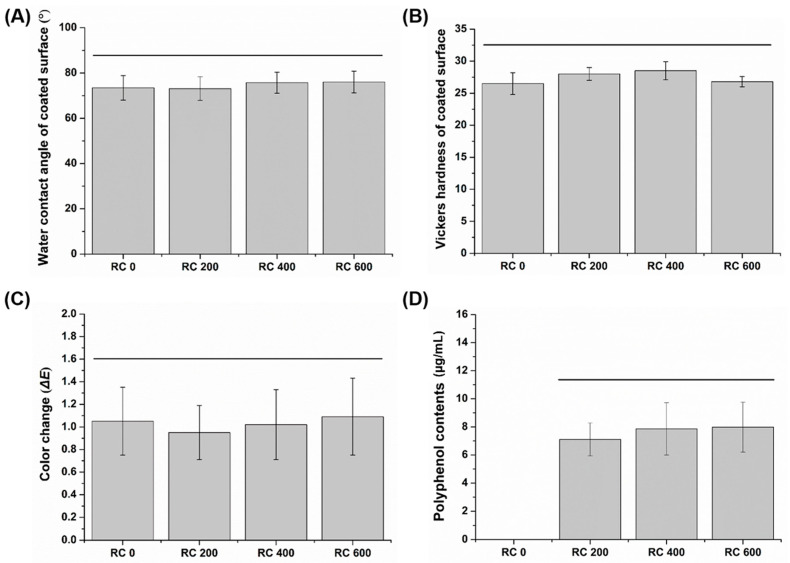
Bar graphs showing the (**A**) water contact angles, (**B**) Vickers microhardness, (**C**) color change before and after the coating treatment of the surfaces of the control and experimental groups, and (**D**) the amount of released polyphenol from the control and experimental groups. Each value shows the average of five measurements, and the error bar indicates the standard deviation of the average. The horizontal bar indicates no significant differences between groups (*p* > 0.05).

All experimental and control groups exhibited no significant differences in the water contact angle values (*p* > 0.05) (Figure 1A). In addition, there was no significant difference in the water contact angle, regardless of different *R. elliptica* extract concentration in the coating resin for the experimental group (*p* > 0.05). Therefore, the *R. elliptica* extract had no significant effect on the surface contact angles. The water contact angles of the coated surface were: 73.5 ± 5.4, 73.1 ± 5.2, 75.7 ± 4.6, and 76.0 ± 4.8 for RC 0, RC 200, RC 400, RC 600, respectively. Additionally, the water contact angle of the uncoated disk specimens was 84.8 ± 6.5, which was significantly higher than control and experimental groups (*p* < 0.05).

### 2.2. Microhardness of the Coated Surface

All the experimental and control groups exhibited no significant difference in microhardness values (*p* > 0.05) (Figure 1B). In addition, within the experimental group, there was no significant difference in the microhardness value regardless of different *R. elliptica* extract concentrations in the coating resin (*p* > 0.05). Therefore, the *R. elliptica* extract exhibited no significant effect on the surface microhardness. The microhardness values of the coated surfaces were: 26.5 ± 1.7, 28.0 ± 1.0, 28.5 ± 1.4, and 26.8 ± 0.8 for RC 0, RC 200, RC 400, and RC 600, respectively. Additionally, the microhardness of the uncoated disk specimens was 10.8 ± 1.2, which was significantly lower than that of the control and experimental groups (*p* < 0.05).

### 2.3. Color Characterization

All the experimental and control groups exhibited no significant difference in the *ΔE* values (*p* > 0.05) (Figure 1C). In addition, the experimental group revealed that there was no significant difference in the *ΔE* value, regardless of different *R. elliptica* extract concentration in the coating resin (*p* > 0.05). Therefore, the *R. elliptica* extract exhibited no significant effect on the surface color. The color difference before and after the coating treatment were: 1.1 ± 0.3, 1.0 ± 0.2, 1.0 ± 0.3, and 1.1 ± 0.3 for RC 0, RC 200, RC 400, and RC 600, respectively.

### 2.4. Analysis of Polyphenol Content

Polyphenol was not detected in the control group (RC 0), but was detected in all the experimental groups. The experimental groups exhibited no significant difference in the polyphenol content, regardless of different *R. elliptica* extract concentration in the coating resin (*p* > 0.05) (Figure 1D). The coating resin containing *R. elliptica* extract had effect on the release of polyphenol. The polyphenol content in the extracted solution from the experimental coated specimens were: 7.11 ± 1.17, 7.86 ± 1.86, and 7.98 ± 1.78 for RC 200, RC 400, RC 600, respectively.

### 2.5. Antimicrobial Activity

The growth inhibitory effect of the control and experimental groups on *S. mutans* and *C. albicans* is shown in Table 1. When considering the control and experimental groups at each culture time point in *S. mutans*, the control group (RC 0) with no *R. elliptica* extract revealed a higher optical density (OD) value than other experimental groups containing *R. elliptica* extract (*p* < 0.05). When considering the control and experimental groups at each culture time point in *C. albicans*, there was no significant difference in the OD values between the control and the experimental groups after 24 h of incubation (*p* > 0.05). However, after 48 h of incubation, the OD values of all the experimental groups were significantly lower than those of the control group (*p* < 0.05). This shows that the coating resin, containing *R. elliptica* extract, significantly impacted the OD value of each microbial, compared with the RC 0 after 48 h (*p* < 0.05). After 48 h of incubation, there was a significant difference between the experimental groups in the OD values of all microorganisms (*p* < 0.05).

**Figure 2:**
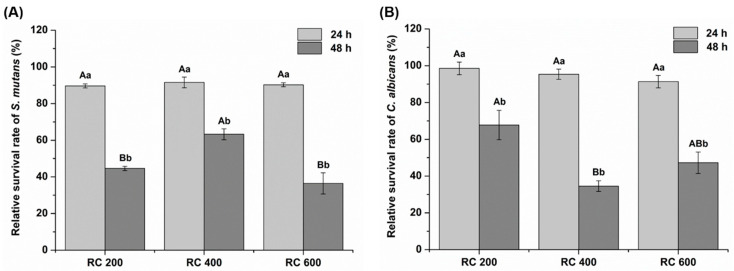
Relative survival rate of (**A**) *Streptococcus mutans;* and (**B**) *Candida albicans* exposed to the extract solution of experimental coating resin, containing *Reynoutria elliptica* extract at two different time points. The same uppercase letters exhibit no significant differences in the survival ratio between the experimental groups at each time point (*p* > 0.05), whereas the different lowercase letters indicate significant differences in the survival ratio between two different time points at each group (*p* < 0.05).

Figure 2 shows the relative microbial survival rate of the experimental groups based on the control coated group (RC 0) that did not contain the *R. elliptica* extract. There was no significant difference in the survival rate between the experimental groups after 24 h in all microorganisms (*p* > 0.05). However, there was a significant difference between the experimental groups after 48 h (*p* < 0.05). In all microorganisms, the survival rate of the microorganisms after 48 h of incubation was significantly lower than that after 24 h of incubation, but not the concentration-dependent trend of *R. elliptica* extract (*p* < 0.05).

### 2.6. Cell Viability

**Figure 3:**
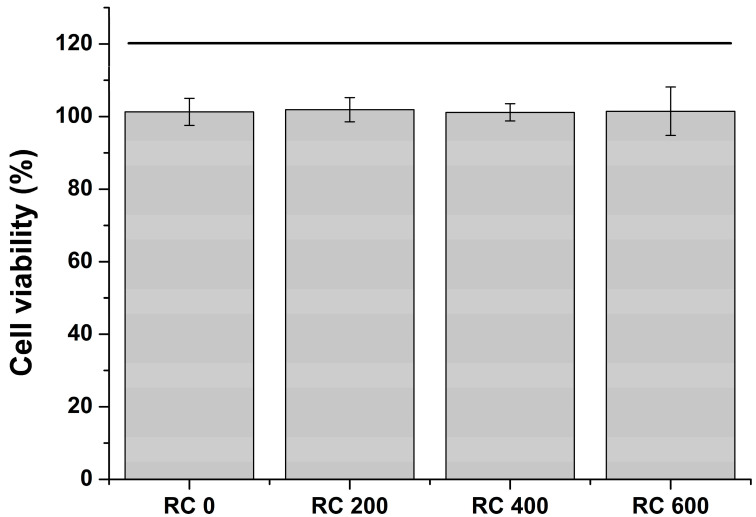
Bar graph showing the cell viability of the extract solutions from the experimental and control groups. Each value shows the average of five measurements, and the error bar indicates the standard deviation of the average. The horizontal bar shows no significant differences among the groups (*p* > 0.05).

All experimental and control groups exhibited no significant difference in the cell viability values (*p* > 0.05) (Figure 3). In addition, the experimental group revealed no significant difference in the cell viability value, regardless of different *R. elliptica* extract concentration in the coating resin (*p* > 0.05). Additionally, the *R. elliptica* extract had no significant effect on cell viability. The cell viability of the experimental and control groups was almost 100%, demonstrating that the extract solutions from all groups were biocompatible.

## 3. Discussion

Applying a coating resin to the denture surface not only improves the mechanical and chemical properties of the material, but also demonstrates beneficial factors, such as biological resistance to oral pathogens and color change resistance [3]. Previous studies on the surface modification of acrylic resin-based materials have been continuously undertaken, postulating the inhibition of growth and the death of oral pathogenic microorganisms [46,47,48,49]. In addition, numerous studies have attempted to inhibit the attachment of microorganisms to material surfaces [50]. However, the synthetic chemicals in other studies exhibited low biocompatibility due to cytotoxicity at high concentrations, although they effectively suppressed the growth of pathogens because of their antibacterial activity on the denture surfaces [51]. Therefore, it is imperative to carefully select the antimicrobial materials because the continued use of synthetic chemicals and antibiotics can cause side effects, such as low cell viability or mutagenicity [45].

Many plant-derived natural medicines have been studied to explore their antibacterial and anti-inflammatory effects. Currently, natural medical ingredients that inhibit the activity of microbes that cause oral disease have been proposed for use in the field of dentistry [42,43]. Hence, in this study, the surface characteristics, antimicrobial activity, and biocompatibility of acrylic resins coated with *R. elliptica* extract were evaluated. In addition, the polyphenol content was examined to analyze the extracts from the coated acrylic resin disk.

In previous studies, the coating treatment demonstrated potential for corrosion protection, good stain resistance, and superior mechanical and chemical properties compared to the group without coating treatment [3]. Accordingly, the coating applied to the surface of the dental material should be chemically stable and crack resistant, without deteriorating the material properties [52,53]. Dental clinicians have stated the importance of retaining close contact with denture base materials and supporting tissues in the oral cavity for the maintenance and stability of removable dentures [54]. One way to improve denture maintenance is by maintaining the wettability of the material surface, i.e., it should not be reduced [55,56]. The contact angle of the coated surface can imply the degree of retention of denture in the oral cavity based on the wettability results to moisture [57]. Therefore, in this study, the contact angle was measured using distilled water, a polar solution, in order to evaluate the wettability of the coating resin containing various concentrations of *Reynoutria elliptica* extract. As shown in Figure 1A, the contact angle of the coated acrylic resin surface was not significantly different among the groups (*p* > 0.05). Moreover, compared to the uncoated group, the experimental and control groups exhibited significantly lower contact angles. Further, it was expected that the retention with oral mucosa could improve when the coated denture base material was applied to the oral cavity. As such, our results imply that the various concentrations of *R. elliptica* extract did not degrade the wettability of the coating resin.

Wear refers to a phenomenon in which material is gradually lost due to mutual mechanical action between a contacting material and a surface. The mechanism that causes wear on the denture surface is very complicated due to the dynamic oral environment, but abrasive wear is generally dominant [58,59,60]. Abrasion resistance correlates with surface hardness, and abrasion proceeds rapidly on the surface of a material with low hardness [61,62]. Abrasion of acrylic resin can lead to the accumulation of food debris and calculus, and the adhesion of bacteria by roughening the surface [63]. Therefore, it is possible to predict the wear resistance by measuring the surface hardness of the material [64]. As shown in Figure 2B, there was no significant difference in the hardness values between the experimental and control groups, indicating a significantly higher hardness than that of the uncoated acrylic resin surface. These results confirmed that the reduction in surface hardness of the acrylic resin material was independent of the concentration of the *R. elliptica* extract. Moreover, coated materials are presumably more resistant to abrasion than uncoated denture materials.

Coating resins containing *R. elliptica* extract should not degrade the aesthetic properties of acrylic resin materials. In this study, we attempted to determine whether the coating treatment can maintain the original color of the acrylic resin material. A spectrophotometer with high accuracy and reproducibility was used for the quantitative comparison of color changes [65]. A before, and after, comparison of the surface treatment using coating resins containing various concentrations of *R. elliptica* (Figure 1C) revealed a low value without significant difference between the control and experimental groups. The color change was difficult to distinguish with the naked eye. Further, the *R. elliptica* extract did not have a significant effect on the color change of the coated surface. Accordingly, based on the results of the surface characterization, the first null hypothesis was accepted, indicating that the surface coating treatment with various concentrations of the *R. elliptica* extract did not significantly impact the water contact angle, hardness, and color of the acrylic denture material surface.

The microbial used in our research are bacteria and fungi, which are most important pathogens in the mouth. *Streptococcus mutans* and *C. albicans* are plaque colonizers and are strongly attached to tooth surfaces or acrylic resin-based denture materials. The antimicrobial activity tests can be divided into methods for completely killing bacteria or fungi and for inhibiting proliferation. Completely killing bacteria may be excellent in terms of effectiveness, but it is more ideal to have bactericidal power that can selectively target on specific microorganisms to prevent killing other normal microbes in the oral cavity. In this study, the growth inhibitory effect of *S. mutans* and *C. albicans* with an extract solution from acrylic resin material coated with various concentrations of the *R. elliptica* extract was assessed. As shown in Figure 2, the experimental groups containing *R. elliptica* extract significantly impacted the growth of *S. mutans* and *C. albicans* (*p* < 0.05). These results show that an acrylic resin material coated with *R. elliptica* extract inhibits the activity of oral pathogenic microbes. In addition, there were significant differences in the optical density values for all groups containing *Reynoutria elliptica* extract after 48 h of cultivation (*p* < 0.05). These results indicate that the experimental groups had a higher growth inhibitory effect than the control group in both *S. mutans* and *C. albicans* after 48 h of cultivation rather than 24 h. Based on the results of the antimicrobial activity, the second null hypothesis was rejected, indicating that the surface coating treatment with different concentrations of the *R. elliptica* extract did not significantly impact the antimicrobial activity against *S. mutans* and *C. albicans*.

As a result of the extract analysis from the coated acrylic resin disk, polyphenol was detected in all the experimental groups coated with the coating resin containing *R. elliptica* extract. These results indicate that the components of the *R. elliptica* extract contained in the coating resins were dissolved and diffused in the liquid environment. The experimental groups revealed that there was, no significant difference in the amount of polyphenol released. Polyphenols, which are chemical substances found in plants, are structured such that one or more phenol groups are bound per molecule. Polyphenols exhibit favorable biological activities such as antioxidants and antibacterial effects [45]. In addition, the antibacterial effect of nature-derived plants is associated with phenolic compounds [66,67]. Therefore, the phenolic compounds released from the *R. elliptica* extract presumably contributed to the inhibition of microbial growth used in this study.

Cytotoxicity testing has limitations in evaluating the systematic review of biocompatibility of materials, but has high reproducibility and is relatively simple. Moreover, it is easy to standardize the test process and obtain quantitative results at low cost over a short period of time. The biological evaluation of dental materials requires a cytotoxicity test as a standard screening procedure [68,69]. In particular, the evaluation of biocompatibility for newly developed materials is essential. After extracting the coated acrylic resins according to international standards, the cytotoxicity of the extracts was evaluated. As a result, there was no significant difference between the control and experimental groups, and both showed a high cell survival rate close to 100%. These results demonstrated that the components extracted from the coating resin were too small or safe to cause cytotoxicity. Based on the findings of the cell viability, the third null hypothesis was accepted, revealing that the surface coating resin containing various concentrations of the *R. elliptica* extract did not significantly impact the viability of L929 fibroblast cells.

We observed whether the surface characterization and antimicrobial activity required for use as a dental material were influenced by adding the plant-derived *R. elliptica* extract to the coating resin. As a result, even though the concentration of the *R. elliptica* extract was lower than that of other antimicrobial materials, it exhibited a growth inhibitory effect on the microbes that cause oral disease. However, since we have only considered the aforementioned over a short duration, it is necessary to further investigate the concentration of the effective antimicrobial activity without changing the coating stability for a prolonged period. Dental acrylic resin material must have clinically acceptable mechanical strength in oral environment. This study confirmed the surface characterization of coated materials, but there was a lack of research on the mechanical strength for pre-clinical investigations. Therefore, the effect of coating treatment on the mechanical properties of acrylic resin materials will also need to be evaluated through further research [27,70]. Despite these limitations, this study evaluated the interaction between experimental materials and microorganisms by simulating the local oral environment in which pathogens exist, in order to evaluate the antimicrobial activity of dental acrylic resins coated with *R. elliptica* extract. In microbial-related oral disease research, our present study is a useful indicator to evaluate the antimicrobial properties of dental materials containing plant extracts [71]. We demonstrated that *R. elliptica* extract can be used as a raw material for antibacterial coating resins. This result will be a meaningful basis for the development of future antimicrobial dental materials using plant-derived extracts.

## 4. Materials and Methods

### 4.1. Coating Resin Containing R. Elliptica Extract

Roots of *R. elliptica*, cultivated in the North Gyeongsang province located in South Korea, were obtained from an herbal shop (Hanyakjae market, Seoul, Korea). The crushed *R. elliptica* specimen (500 g) was immersed in a 70% methanol solution (5 L), and extracted at 25 ± 1 °C for 48 h. The extracted solution was filtered using a filter paper (Grade 2, Whatman^®^, Maidstone, UK). The solution was then concentrated using a vacuum evaporator (EYELA, Tokyo, Japan). The dried powder was obtained by freezing the *R. elliptica* extract at −20 °C for one day, then placing it in a freeze-drying machine (Ilshin Lab, Gyeonggi-do, Korea) at −55 °C for 2 d. Then, the dried *R. elliptica* extract was ground using a mortar and pestle to produce fine particles. The dried *R. elliptica* extract was maintained by storing the obtained fine particles in a desiccator at 25 ± 1 °C before mixing in the coating resin.

The experimental coating resins were prepared by dissolving the *R. elliptica* extract powder in surface coating resin (Plaquit, Dreve, Unna, Germany) at 25 ± 1 °C using a magnetic stirrer in a dark environment for 24 h. Concentrations of 200, 400, and 600 μg/mL were obtained for RC 200, RC 400, and RC 600, respectively. In addition, a surface coating resin that did not contain the *R. elliptica* extract powder was also prepared as previously described to make a control coating resin (RC 0).

### 4.2. Coating Treatment on Acrylic Resin Disks

The Teflon mold (diameter: 10.0 mm, height: 1.0 mm) was placed onto a polyester film on a microscope slide (Paul Marienfeld GmbH, Bad Mergentheim, Germany). The powder and liquid materials of the autopolymerizing acrylic resin product (Jet denture repair, Lang Dental, Wheeling, IL, USA) were mixed according to the instructions from the manufacturer until they showed a dough stage and packed into the Teflon mold to avoid the formation of air bubbles. A polyester film was then placed onto the autopolymerizing acrylic resin and covered with another microscope slide. A clamp was used to apply pressure to the microscope slide and Teflon mold for one minute to displace the excess material. After autopolymerizing for 30 min, the disk-shaped specimens were carefully separated from the mold and any flash on the disk specimen was carefully abraded with 320 grit abrasive paper.

The disk specimens were coated with the control and experimental coating resins with various *R. elliptica* extract concentrations (10 µL) to the disk specimens using a micropipette and micro brush. The specimens were continuously light-cured under vacuum for 10 min using the Visio Beta Vario unit (3M ESPE, Red Wing, MN, USA). This coating treatment was performed on only one side per specimen, and the other side was not coated.

### 4.3. Surface Characterization

To confirm the wettability of the coated control and experimental disk specimens, an optical wettability inspection was performed using a contact angle measuring device (Phoenix 300, SEO, Gyeonggi-do, Korea), combining CCD color cameras and image analysis software. A micro syringe was used to transfer a 5 µL volume of distilled water on the coated surfaces. The water contact angles were recorded 3 s after the drop was deposited. The water contact angle of each droplet was measured at three random points on the coated surfaces, and the average was recorded as the water contact angle of one specimen. The water contact angles were measured at 25 ± 1 °C and 45 ± 5% relative humidity. Additionally, the water contact angles of the uncoated disk specimens were also evaluated.

The microhardness of the coated control and experimental disk specimens was evaluated by measuring the Vickers microhardness at 0.09 MPa for 20 s using a microhardness device (DMH-2, Matsuzawa Seiki Co., Tokyo, Japan). Indentations were made using a diamond indenter. Three measurements were conducted at random points on the surface of the coated specimen and the average value was recorded as the surface microhardness of the specimen. The microhardness of the uncoated disk specimens was also evaluated using the same procedure.

The effect of the experimental coating resin on the color of the acrylic resin disk surface was considered by observing the color of the experimental coated specimens with a spectrophotometer (Lamba20, Perkin Elmer, Orwalk, CT, USA). The *L*, *a*, and *b* values and the color change (*ΔE*) were confirmed according to the CIE color coordinate system. The *L*, *a*, and *b* value reveal lightness or darkness, redness or greenness, and yellowness, or blueness, respectively. The color difference before and after the coating treatment was calculated using the following equation: *ΔE* = ((*ΔL*)^2^ + (*Δa*)^2^ + (*Δb*)^2^)^1/2^ to confirm the color change.

Surface characterization was independently performed with five repetitive tests, and data were recorded as averages and standard deviations.

### 4.4. Analysis of the Coated Sample Extracts

To analyze the extracts from the experimental sample, the coated disk specimens were extracted in distilled water for 7 d at 37 °C. Based on ISO 10993-12, the extraction ratio was 3 cm^2^/mL (surface area of coated disk specimen/distilled water volume) [72]. The extracted polyphenol content in distilled water (experimental coated disk specimen) was quantified by mixing the extracted solution (50 μL) and Folin-Denis’ reagent (50 μL, Sigma Aldrich, St Louis, MO, USA) in distilled water (650 µL), and reacting at 25 ± 1 °C for 3 min. Distilled water (150 µL) and 10% Na_2_CO_3_ solution (100 μL) were mixed to the reacted solution to form a total volume of 1 mL. Subsequently, the treated solution was placed at 37 ± 1 °C in a light-blocked condition. After 60 min, the absorbance value was detected using a UV/VIS spectrometer (X-ma 1200 spectrophotometer, Human Corporation, Seoul, Korea) at 725 nm. Garlic acid (Sigma Aldrich, St Louis, MO, USA) was used as a standard solution to generate the calibration curve (10, 20, and 30 μg/mL). Based on the calibration curve of the standard solution, the total polyphenol content in the extracted solution was estimated in micrograms of garlic acid equivalents. The test for the extract analysis of the coated sample was independently performed with five repetitive tests, and data were recorded as averages and standard deviations.

### 4.5. Antimicrobial Test

To confirm the antimicrobial activity of the coated experimental disk specimens, *S. mutans* (ATCC 25175) and *C. albicans* (ATCC 10231) were used as the test microorganisms. *Streptococcus mutans* and *C. albicans* were cultured in each culture medium, brain heart infusion (BHI, Becton Dickinson and Co., Baltimore County, MD, USA), and yeast mold (YM, Becton Dickinson and Co., Franklin Lakes, NJ, USA), respectively, then incubated at 37 ± 1 °C for 1 d.

The coated experimental disk specimens were extracted in phosphate-buffered saline (PBS, Welgene, Gyeongsangbuk-do, Korea) for 24 h at 37 ± 1 °C. Based on ISO 10993-12, the extraction ratio was 3 cm^2^/mL (surface area of the coated disk specimen/distilled water volume) [72]. In addition, the coated disk specimens that did not contain the *R. elliptica* extract were also extracted under the previously described conditions. This was used as a control in the antimicrobial test.

The antimicrobial activity of the extracts from the coated samples were confirmed by diluting each microbial suspension with culture solution to standardize the OD value (0.4–0.6 at 600 nm). The extract solutions from the coated samples and microbial suspensions were mixed in a 9:1 ratio and incubated at 37 ± 1 °C for 24 and 48 h. The extract solution from the control specimen and microbial suspension was also cultured under the same conditions; this was denoted as the control group. Thereafter, the growth inhibitory efficacy of the each coated sample was determined by measuring the OD values at 600 nm at two different time points using an ELISA reader (Epoch, BioTek, Winooski, VT, USA). In addition, the relative survival rate of each microbial exposed to the extract solution of the experimental coating resin containing *R. elliptica* extract was calculated using the following equation: Microbial survival rate (%) = (Optical density of the experimental coated sample/Optical density of the control coated sample) × 100.

Antimicrobial effect tests were independently conducted with five repetitive tests, and the obtained data were recorded as averages and standard deviations.

### 4.6. Cell Viability Test

Tests for the in vitro biocompatibility test for coated experimental samples were conducted according to the ISO 10993-5 using a 3-[4,5-dimethylthiazol-2-yl]-2, 5 diphenyltetrazolium bromide (MTT) assay [68]. The coated control and experimental samples were maintained for 30 min under ultraviolet (UV) light to prevent contamination. The coated control and experimental samples were then extracted in RPMI 1640 (Gibco Laboratories, Grand Island, NY, USA) cell culture medium containing 10% (*v*/*v*) fetal bovine serum (FBS, Gibco Laboratories, Grand Island, NY, USA) at 37 ± 1 °C in a 5% CO_2_ humidified air condition for 24 h according to ISO standard 10993-12. The extraction ratio was 3 cm^2^/mL (surface area of coated disk specimen/RPMI 1640 cell culture medium containing 10% (*v*/*v*) FBS) [72]. The extraction vehicle without the specimens (blank) was stored under the previously described conditions; this was utilized as a negative control in the cell viability test.

Immortalized L929 mouse fibroblast cells were selected and cultured in RPMI 1640 with 1% antibiotic-antimycotic solution (Anti-Anti, Gibco Laboratories, Grand Island, NY, USA) and 10% (*v*/*v*) FBS, sub-cultured three times in 7 d, at 37 ± 1 °C in a 5% CO_2_ humidified air condition. The adherent cells on the cell culture flask were detached using a mixture of 0.05% Trypsin-EDTA (Gibco Laboratories, Grand Island, NY, USA) stored for 10 min at 37 ± 1 °C. Immortalized L929 mouse fibroblast cells (100 µL, 1 × 10^5^ cells/mL) was seeded on 96-well culture plates (Thermo Scientific, Hanover Park, IL, USA) and stored at 37 ± 1 °C in a 5% CO_2_ humidified air condition for 24 h. One-hundred microliter of the experimental and negative extract solutions were added to each well and further incubated for 24 h. The experimental and negative extract solutions in the 96-well plates were removed from the wells and refilled with 50 μL of 1 mg/mL MTT-tetrazolium salts (Sigma, St. Louis, MO, USA) in PBS. The plates were stored at 37 ± 1 °C in a 5% CO_2_ humidified air condition for 2 h in a light-blocked environment. Then, the MTT solution was removed from the wells and refilled with isopropanol (100 μL, Sigma, St. Louis, MO, USA). The 96-well culture plate was then placed on a shaker for 20 min in a light-blocked condition and transferred to a microplate reader (Epoch, BioTek, Winooski, VT, USA) equipped with a 570 nm filter to read the absorbance. The cell viability in the negative control group (blank) was considered as 100%, and the percentage values for extracts of the control and experimental coated samples were estimated. To calculate the reduction in viability compared to the blank, the following equation was used: Cell viability (%) = (Optical density for extracts of the experimental and control coated samples/Optical density of the blank) × 100. The MTT test was independently performed with five repetitive tests, and the data were recorded as averages and standard deviations.

### 4.7. Statistical Analysis

The surface characterization, extract analysis, antimicrobial activity, and cell viability results from the experimental and control groups did meet the normality in the Kolmogorov-Smirnov test and homoscedasticity in Levenes test, hence differences were analyzed with one-way ANOVA (PASW 18.0, IBM Co., NY, USA) to confirm the interactions between the experimental coating resins with various concentrations of *R. elliptica* extract. Post-hoc analyses were performed by Tukey’s multiple comparison test at a significance level of 0.05 to determine the significance at different concentrations.

## 5. Conclusions

In the present study, the *R. elliptica* extract incorporated surface coating resin was fabricated and evaluated in terms of surface characterization, antimicrobial activity, and biocompatibility. The results revealed that the *R. elliptica* extract did not impact the water contact angle, hardness or color of the acrylic resin material surface. However, the antimicrobial activity test revealed that the *R. elliptica* extract impacted the antimicrobial activity against *S. mutans* and *C. albicans* strains, which are oral pathogens. More importantly, the *R. elliptica* extract did not affect cell viability.

In brief, the results of this study suggest that coating treatment with plant-derived *R. elliptica* extract can be applied in dental acrylic resin materials with antimicrobial properties to prevent oral diseases, with no deterioration of the surface characteristics and biocompatibility.

## Figures and Tables

**Table 1:** Optical density value of the control and experimental groups with *Streptococcus mutans* and *Candida albicans* after 24 and 48 h of cultivation.

Experimental Groups	*Streptococcus Mutans*		*Candida Albicans*	
	24 h	48 h	24 h	48 h
RC 0	0.053 ± 0.003 ^a^	0.612 ± 0.008 ^a^	0.042 ± 0.002 ^a^	0.829 ± 0.049 ^a^
RC 200	0.047 ± 0.001 ^b^	0.273 ± 0.007 ^c^	0.041 ± 0.001 ^a^	0.599 ± 0.071 ^b^
RC 400	0.048 ± 0.002 ^b^	0.387 ± 0.018 ^b^	0.040 ± 0.001 ^a^	0.305 ± 0.025 ^c^
RC 600	0.048 ± 0.001 ^b^	0.223 ± 0.035 ^c^	0.038 ± 0.001 ^a^	0.418 ± 0.052 ^c^

Different superscript letters show significant differences between the control and experimental groups at each cultivation time (*p* < 0.05).
